# First-Borns Carry a Higher Metabolic Risk in Early Adulthood: Evidence from a Prospective Cohort Study

**DOI:** 10.1371/journal.pone.0013907

**Published:** 2010-11-09

**Authors:** Mario Siervo, Bernardo L. Horta, Blossom C. M. Stephan, Cesar G. Victora, Jonathan C. K. Wells

**Affiliations:** 1 Human Nutrition and Physiology, Department of Neuroscience, University “Federico II”, Naples, Italy; 2 Postgraduate Program in Epidemiology, Federal University of Pelotas, Pelotas, Brazil; 3 Institute of Public Health, University of Cambridge, Cambridge, United Kingdom; 4 Childhood Nutrition Research Centre, UCL Institute of Child Health, London, United Kingdom; Institute of Preventive Medicine, Denmark

## Abstract

**Background:**

Birth order has been associated with early growth variability and subsequent increased adiposity, but the consequent effects of increased fat mass on metabolic risk during adulthood have not been assessed. We aimed to quantify the metabolic risk in young adulthood of being first-born relative to those born second or subsequently.

**Methodology and Principal Findings:**

Body composition and metabolic risk were assessed in 2,249 men, aged 17–19 years, from a birth cohort in southern Brazil. Metabolic risk was assessed using a composite z-score integrating standardized measurements of blood pressure, total cholesterol, high density lipoprotein, triglycerides and fat mass. First-borns had lower birth weight z-score (Δ = −0.25, 95%CI −0.35, −0.15,p<0.001) but showed greater weight gain during infancy (change in weight z-score from birth to 20 months: Δ = 0.39, 95%CI 0.28–0.50, p<0.0001) and had greater mean height (Δ = 1.2 cm, 95%CI: 0.7–1.6, p<0.0001) and weight (Δ = 0.34 kg, 95%CI: 0.13–0.55, p<0.002) at 43 months. This greater weight and height tracked into early adulthood, with first-borns being significantly taller, heavier and with significantly higher fat mass than later-borns. The metabolic risk z-score was significantly higher in first-borns.

**Conclusions/Significance:**

First-born status is associated with significantly elevated adiposity and metabolic risk in young adult men in Brazil. Our results, linking cardiovascular risk with life history variables, suggest that metabolic risk may be associated with the worldwide trend to smaller family size and it may interact with changes in behavioural or environmental risk factors.

## Introduction

The metabolic syndrome is a key factor contributing to morbidity and mortality worldwide, both in industrialised populations and those passing through the nutritional transition [1]. Rising levels of obesity account for a significant proportion of the increase in prevalence of the metabolic syndrome [2]. Public health efforts to reduce risk factors associated with the increase in obesity are therefore a priority for global health.

However, not all risk factors for obesity are readily modifiable. Dietary energy density is an example of a modifiable determinant, as a propensity to consume energy-dense foods, associated with weight gain [3], can be countered by a range of policies acting either on individual behaviour or the food industry [4]. Non-modifiable risk factors, such as genetic polymorphisms (MC4R,FTO) [5] or gender, cannot by definition be altered though the condition can still of course be treated. Nonetheless, identification of the impact of such factors is critical for the development of targeted public health or pharmacological interventions aimed at limiting their effect on obesity risk, and for understanding the likely impact of interventions on modifiable factors.

In this context is it valuable to investigate life history variables. Many life history traits such as age at maturation and adult size are related to early growth patterns [6], [7], which in turn have been associated in many studies with metabolic risk [8], [9]. However, the majority of biomedical studies of early growth variability have focused on clinical factors such as maternal pregnancy weight gain, intra-uterine growth retardation, maternal smoking or preterm birth [7], [10], [11], [12].

Recent work has suggested that birth order may be a non-modifiable risk factor for obesity. Current evidence suggests that first-born infants grow faster than later-born infants[10]. Dunger et al.[13] suggest that the *in-utero* growth of first-born babies may be restrained as they have lower birth weight and accelerated post-natal catch-up growth [10], both of which are risk factors for obesity [14] and cardiovascular and metabolic diseases [15], [16] in adult life. However, whether first-born individuals have elevated metabolic risk in adulthood remains unknown. A recent study found that first-borns had a 4-fold risk of increased fat mass in early adulthood compared to later-borns [17]. Neither of these studies evaluated the magnitude of metabolic risk induced by such greater weight and adiposity.

Identification of the effect of birth order on metabolic risk is important in relation to demographic trends, such as the restrictive family planning policies (one child policy) adopted in some nations and the worldwide decline in fertility [18], [19], [20].

Here we investigate the associations of birth-order with metabolic phenotype in early adulthood using data from a birth cohort of Brazilian young men. We tested two hypotheses. First, we wanted to confirm that first-born status was associated with low birth weight and faster infant growth. Second, we tested the hypothesis that metabolic risk was increased in first-borns compared to later-borns.

## Methods

### Subjects and Protocol

During 1982, the three maternity hospitals in Pelotas, a southern Brazilian city, were visited daily and the 5914 live born infants whose family lived in the urban area of the city were weighed and their mothers interviewed. These children have been followed up on a number of occasions [21]. In 1984 and 1986, all household (approximately 70000) in the city were visited in search of children born in 1982; this approach led to tracing 87% and 84% of the original cohort, respectively. In all visits, subjects were weighed with calibrated scales, and their height was measured using portable stadiometers. In 2000, all males in the birth cohort who were still living in the city were legally obliged to take part in an examination at the local army base. Those who agreed signed a detailed informed consent form and underwent the physical examination; 79% of all males in the original cohort were traced. The study was approved by the Research Ethics Committee of the Faculty of Medicine, Federal University of Pelotas (affiliated with the Brazilian National Council of Research Ethics) and all subjects provided written informed consent.

Socio-demographic and lifestyle information were collected, including: 1) schooling (1–4, 5–8 or 9–12 years); 2) social status (single, married); 3) smoking history (yes, no); 4) birth order rank (first-born, later-born); and 5) regular physical activity (yes, no). Information was also collected in the early cohort visits on family income, maternal schooling, household wealth score and maternal smoking status during pregnancy, duration of breastfeeding.

### Anthropometry, Body Composition

Standing height was measured by a CMS stadiometer to the nearest mm with subjects barefood. Subjects were weighed in minimal clothing using a Tanita Body Fat Analyser scale (model TBF-305; Japan), which also provided information on body composition through bio-electrical impedance. A validation sub-study was conducted in sample of 48 participants in the age range of the study cohort using total body water through deuterium dilution as the gold standard. We used the resulting validation equation (total body water  = 4.437+(0.378×weight)+((0.189×height^2^)/impedance) to calculate fat-free mass (FFM, calculated as total body water/0.732) and hence fat mass (FM) as the difference from weight. Body mass index (BMI) was calculated and subjects categorised into three weight categories based on WHO cut-off scores including: normal weight (18.5<BMI<25 kg/m^2^), overweight (25≤BMI<30 kg/m^2^) and obese (BMI≥30 kg/m^2^). Body composition data were adjusted for height to calculate the fat mass index (FMI (kg/m^2^) = FM divided by height^2^) and fat free mass index (FFMI (kg/m^2^) = FFM divided by height^2^)[22]. These adjust FFM and FM for body size independently of one another. The FM:FFM ratio was also calculated. Maternal height and weight were measured at the beginning of the pregnancy. Weight and stature of the children was measured at birth and at each follow up visit. Birth weight z scores were computed using the following formula: z = (x−mean)/SD, where x is the infant's birth weight and mean and SD are the mean and standard deviation for each gestational age and sex group in the reference population [23]. Using the NCHS growth reference[24], z scores were also calculated for weight adjusted for age in the follow up visits. Changes in body size (weight z score, crude weight and height) between birth and the follow up visits were calculated.

### Clinical Biochemistry

During the Army interview the cohort members were invited to donate a blood sample. Typically, conscripts had a continental-style breakfast at home at around 5:30 am, because they had to arrive at 6:00 am at the Army Base where the exams were carried out. Blood samples were collected by venepuncture between 10:30am and 12:00 noon. Total and HDL cholesterol and triglycerides were measured using enzymatic methods. LDL cholesterol was estimated using Friedewald's formula[25]. The shorter post-prandial period may have interfered with the measurements of some blood parameters, particularly lipid profile. However, serum lipid levels were similar to results from other Latin American settings measuring metabolic parameters after a conventional 12 hours fasting. This indicates an overall representativeness of the blood tests. In addition, it could be argued that the potential measurement bias would introduce a random rather than a differential error and therefore unlikely to affect the direction of the relationship between birth-order groups.

### Statistical Analysis

Continuous variables were described using summary statistics. Student's t-test for independent samples was used to detect differences between subjects categorised according to birth order (first-born versus later-born). The chi square (χ^2^) test was used for the categorical variables. The sample size was sufficient to detect a difference between birth order groups of 0.15 z-scores.

A continuous metabolic risk z-score was computed as the average of the z-scores for the individual traits, to evaluate differences in risk between first- and later-born subjects[26]. The risk z-score was calculated using the following variables: FM, HDL, LDL, triglycerides and systolic and diastolic blood pressure. The individual z-score was reversed for HDL to indicate a higher metabolic risk with decreasing values. Crude and adjusted linear regression analyses between birth order and metabolic parameters (including body composition, clinical biochemistry and metabolic risk z-score) were performed. The analysis was adjusted for family income, maternal education, household wealth score, breastfeeding for at least six months, maternal smoking during pregnancy, maternal weight at the beginning of the pregnancy, maternal height, and subject smoking at 18 years. Metabolic risk z-score was also adjusted for potential mediating factors including birth weight z-score and weight gain z-score between birth and 20 months. The same analysis was conducted after excluding 683 only children to exclude the possibility that birth order effects arose from family size effects. SPSS 16 software (SPSS for Windows, SPSS Inc, USA) was used for the statistical analysis. The significance cut-off value was taken at 0.05.

## Results

### Demographics

Birth order groups did not differ significantly for the subjects' age. Achieved schooling was higher among first-borns, whereas the prevalence of tobacco smoking was higher among later-borns. Groups did not differ significantly for physical activity level, household wealth score and marital status. Maternal smoking and breastfeeding for at least 6 months were not different between the two groups, but maternal schooling, maternal weight and the proportion of underweight mothers at the beginning of the pregnancy were higher in first-borns (**Table 1**).

**Table 1:** Baseline characteristics of study participants by birth order status in Brazilian sample.

	First-born(N = 917)	Later-born(N = 1332)	p value
**Socio-Demographic**			
Age (years)	18.2 (0.3)	18.2 (0.3)	0.06
Current Smoker, n (%)	112 (12.3)	242 (18.2)	**<0.001**
Achieved schooling in years, n(%)1–45–89–12	57 (6.3)445 (48.9)408 (44.8)	156 (11.9)764 (58.3)391 (29.8)	**<0.001**
Married, n (%)	35 (3.8)	64 (4.8)	0.26
Physically Active, n (%)	172 (18.8)	230 (17.3)	0.36
Family income at birth minimum wages≤11.1–33.1–6>6	20.747.720.411.2	20.050.118.211.7	0.50
Maternal schooling in years0–45–89–11≥12	23.846.713.416.1	37.842.18.411.7	**<0.001**
Household wealth score1^st^ quartile2^nd^ quartile3^rd^ quartile4^th^ quartile	26.127.224.722.0	25.524.724.924.9	0.36
Mother smoked during pregnancy (%)	33.6	35.6	0.30
Maternal weight at the beginning of the pregnancy (kg)	53.9 (8.4)	57.5 (10.5)	**<0.001**
Maternal height (cm)	156.9 (6.4)	156.3 (5.9)	**0.02**
Breastfeeding for at least 6 months (%)	27.4	29.1	0.36
**Body Composition**			
Weight (kg)	68.1 (13.2)	66.7 (12.3)	**0.01**
Height (cm)	173.9 (6.7)	173.1 (6.8)	**0.006**
BMI (kg/m^2^)	22.5 (3.9)	22.2 (3.6)	0.15
FM (kg)	11.7 (5.1)	11.1 (4.7)	**0.009**
FMI (kg/m^2^)	3.8 (1.6)	3.7 (1.5)	**0.03**
FFM (kg)	56.6 (8.5)	55.7 (8.0)	**0.01**
FFMI (kg/m^2^)	18.7 (2.4)	18.6 (2.3)	0.24
**Blood Pressure**			
Systolic (mmHg)	134.5 (14.1)	134.8 (14.1)	0.64
Diastolic (mmHg)	76.3 (12.1)	75.9 (11.9)	0.45
**Biochemistry**			
T-CHO (mmol/L)	3.7 (0.7)	3.6 (0.7)	**0.03**
HDL (mmol/L)	1.0 (0.2)	1.0 (0.2)	0.98
LDL (mmol/L)	2.3 (0.6)	2.2 (0.6)	**0.04**
TRI (mmol/L)	0.8 (0.5)	0.8 (0.5)	0.20
Metabolic risk z-score	0.07 (0.50)	0.01 (0.48)	**0.01**

N =  number of subjects. FM =  fat mass; FFM =  fat free mass; Fat free mass index (FFMI)  = FFM (kg)/height^2^ (m); Fat mass index (FMI)  = FM (kg)/height^2^ (m); T-CHO =  total cholesterol; HDL =  high density lipoproteins; TRI =  triglycerides.

T-test for independent samples was used to compare the two groups. Data are mean (SD), unless otherwise indicated.

The calculation of the metabolic risk z-score is described in the methods section.

### Growth patterns and body composition

After adjusting for family income, maternal education, household assets score and maternal smoking in pregnancy, first-borns had significantly lower mean birth weight z-score (Δ = −0.25, 95%CI −0.35, −0.15, p<0.0001). First-borns also showed faster weight gains during infancy (change in weight z-score from birth to 20 months: Δ = 0.39, 95%CI 0.28, 0.50, p<0.0001) and had greater mean height (Δ = 1.2 cm, 95%CI: 0.7, 1.6, p<0.0001) and weight (Δ = 0.34 kg, 95%CI: 0.13, 0.55, p<0.002) at 43 months (data not shown). This greater weight and height tracked into early adulthood, with first-borns being significantly taller and heavier than later-borns. Although BMI was not related to birth order, first-borns had a significantly higher adiposity (FM) compared to later-borns (**Table 1**).

### Metabolic risk

Total cholesterol and low-density lipoproteins were higher among first-borns. On the other hand, first and later-borns presented similar blood pressure. The metabolic risk z-score was significantly higher in first-borns (**Table 1**)**.** The adjusted regression analysis showed the independent effect of birth order on body composition and metabolic risk. First-borns had higher body weight (Δ = 2.16 kg; 95%CI: 1.08, 3.24,p = 0.001), FMI (Δ = 0.23 kg; 95%CI: 0.09, 0.37,p = 0.001), FFMI (Δ = 0.31 kg; 95%CI: 0.10, 0.52,p = 0.004),fat mass/fat free mass ratio (Δ = 0.01; 95%CI 0.003, 0.01,p = 0.001), BMI (Δ = 0.53 kg; 95%CI: 0.19, 0.86, p = 0.002), triglycerides (Δ = 0.05 kg; 95%CI: 0.002, 0.10, p = 0.04), and metabolic risk z-score (Δ = 0.08, 95%CI: 0.03, 0.13,p = 0.001) compared to later-borns (**Table 2**)**.** The exclusion of only children from the analysis magnified the effects of first-born status on body composition and metabolic risk (**Table 3**). **Table 4** shows that the addition of birth weight z-score to the model did not remove the effect of birth order on metabolic risk (Model 3) but the effect was slightly reduced (from Δ = 0.06, 95%CI: 0.01, 0.11 to Δ = 0.05, 95%CI: −0.007; 0.1) and lost statistical significance when infant weight gain between birth and 20 months was added to the model (Model 4). Similarly, the exclusion of first-born children with status of only children from the analysis did not modify the effect of birth weight and weight gain on the association between birth order and metabolic risk; only significance level was reduced due to smaller power of the analysis (**Table 5**).

**Table 2:** Crude and adjusted linear regression analysis illustrating effect of first-born status on metabolic and body composition parameters.

	Unadjusted B (±95%CI)	Adjusted B (±95%CI)
Height(cm)	0.81 (0.24; 1.38)***p = 0.01***	0.71 (0.15; 1.26)***p = 0.01***
Weight(kg)	1.35 (0.28; 2.43)***p = 0.01***	2.16 (1.08; 3.24)***p = 0.001***
Fat Mass/Height^2^(kg/m^2^)	0.15 (0.01; 0.28)***p = 0.03***	0.23 (0.09; 0.37)***p = 0.001***
Fat Free Mass/Height^2^ (kg/m^2^)	0.12 (−0.08; 0.32)*p = 0.24*	0.31 (0.10; 0.52)***p = 0.004***
Fat Mass/Fat Free Mass	0.01 (0.001; 0.01)***p = 0.01***	0.01 (0.003; 0.01)***p = 0.001***
Body Mass Index(kg/m^2^)	0.23 (−0.08; 0.55)*p = 0.15*	0.53 (0.19; 0.86)***p = 0.002***
Systolic blood pressure(mmHg)	−0.29 (−1.48; 0.91)*p = 0.64*	−0.33 (−1.62; 0.95)*p = 0.61*
Diastolic blood pressure(mmHg)	0.39 (−0.62; 1.40)*p = 0.45*	0.28 (−0.80; 1.36)*p = 0.61*
Total Cholesterol(mmol/L)	0.07 (0.005; 0.13)***p = 0.03***	0.06 (−0.006; 0.13)*p = 0.08*
HDL Cholesterol(mmol/L)	0.0002 (−0.02; 0.02)*p = 0.98*	0.001 (−0.02; 0.02)*p = 0.94*
LDL Cholesterol(mmol/L)	0.05 (0.0007; 0.11)*p = 0.05*	0.04 (−0.02;0.10)*p = 0.22*
Triglycerides(mmol/L)	0.03 (−0.01; 0.07)*p = 0.21*	0.05 (0.002; 0.10)***p = 0.04***
Metabolic risk z-score	0.06 (0.01; 0.11)***p = 0.01***	0.08 (0.03; 0.13)***p = 0.001***

B =  regression coefficient for first-borns; ±95CI  = 95% Confidence Interval.

Significant p values are shown in bold.

Brazilian cohort: Analysis was adjusted for family income; maternal education; household wealth score, breastfeeding for at least six months, maternal smoking during pregnancy, maternal weight at the beginning of the pregnancy, maternal height, and subject smoking at 18 years.

The calculation of the metabolic risk z-score is described in the methods section.

**Table 3:** Crude and adjusted linear regression analysis illustrating effect of first-born status on metabolic and body composition parameters – Excluding first-born children with status of only children.

	Unadjusted B (±95%CI)	Adjusted B (±95%CI)
Height(cm)	1.37 (0.42; 2.32)***p = 0.005***	1.38 (0.45; 2.30)***p = 0.004***
Weight(kg)	1.90 (0.18; 3.63)***p = 0.03***	2.97 (1.24; 4.70)***p = 0.001***
Fat Mass/Height^2^(kg/m^2^)	0.20 (−0.01; 0.41)***p = 0.07***	0.32 (0.10; 0.54)***p = 0.004***
Fat Free Mass/Height^2^ (kg/m^2^)	0.08 (−0.24; 0.41)*p = 0.61*	0.31 (−0.03; 0.64)*p = 0.074*
Fat Mass/Fat Free Mass	0.01 (0.001; 0.02)***p = 0.02***	0.01 (0.005; 0.02)***p = 0.002***
Body Mass Index(kg/m^2^)	0.28 (−0.23; 0.79)*p = 0.28*	0.63 (0.10; 1.16)***p = 0.02***
Systolic blood pressure(mmHg)	0.68 (−1.31; 2.68)*p = 0.50*	0.80 (−1.31; 2.91)*p = 0.46*
Diastolic blood pressure(mmHg)	0.44 (−1.22; 2.10)*p = 0.60*	0.47 (−1.27; 2.21)*p = 0.60*
Total Cholesterol(mmol/L)	0.07 (−0.04; 0.18)*p = 0.23*	0.06 (−0.06; 0.17)*p = 0.34*
HDL Cholesterol(mmol/L)	−0.009 (−0.05; 0.03)*p = 0.66*	−0.009 (−0.05; 0.03)*p = 0.64*
LDL Cholesterol(mmol/L)	0.05 (−0.05; 0.14)*p = 0.35*	0.02 (−0.08;0.12)*p = 0.69*
Triglycerides(mmol/L)	0.06 (−0.02; 0.14)*p = 0.12*	0.09 (0.01; 0.18)***p = 0.02***
Metabolic risk z-score	0.07 (−0.004; 0.15)*p = 0.06*	0.09 (0.02; 0.17)***p = 0.02***

B =  regression coefficient for first-borns; ±95CI  = 95% Confidence Interval.

Significant p values are shown in bold.

Brazilian cohort: Analysis was adjusted for family income; maternal education; household wealth score, maternal smoking during pregnancy, breastfeeding for at least six months, maternal weight at the beginning of the pregnancy, maternal height, and subject smoking at 18 years.

The calculation of the metabolic risk z-score is described in the methods section.

**Table 4:** Brazilian cohort: crude and adjusted linear regression analysis to investigate the prediction of metabolic risk z-score by birth order and explore the effects of birth weight and catch up growth after 20 months.

	Model 1	Model 2	Model 3	Model 4
Metabolic risk z-score	0.06 (0.01; 0.11)***p = 0.01***	0.08 (0.03; 0.13)***p = 0.001***	0.08 (0.03; 0.13)***p = 0.003***	0.05 (−0.0002; 0.1)*p = 0.051*

B =  regression coefficient for first-borns; ±95CI  = 95% Confidence Interval. Significant p values are shown in bold.

Brazilian Cohort: Analysis was adjusted as follows:

Model 1: Unadjusted.

Model 2: Adjusted for family income; maternal education; household wealth score; breastfeeding for at least six months; maternal smoking during pregnancy; maternal weight at the beginning of the pregnancy, maternal height, and subject smoking at 18 years.

Model 3: Adjusted for model 2+ birth weight z-score.

Model 4: Adjusted for model 3+ weight gain z-score birth to 20 months.

The calculation of the metabolic risk z-score is described in the methods section.

**Table 5:** Brazilian cohort: crude and adjusted linear regression analysis to investigate the prediction of metabolic risk z-score by birth order and explore the effects of birth weight and catch up growth after 20 months – Exclusion of first-born children with status of only children.

	Model 1	Model 2	Model 3	Model 4
Metabolic risk z-score	0.07 (−0.004; 0.15)*p = 0.06*	0.09 (0.02; 0.17)***p = 0.02***	0.08 (−0.006; 0.16)*p = 0.07*	0.04 (−0.04; 0.12)*p = 0.31*

B =  regression coefficient for first-borns; ±95CI  = 95% Confidence Interval. Significant p values are shown in bold.

Brazilian Cohort: Analysis was adjusted as follows:

Model 1: Unadjusted.

Model 2: Adjusted for family income; maternal education; household wealth score; breastfeeding for at least six months; maternal smoking during pregnancy; maternal weight at the beginning of the pregnancy, maternal height, and subject smoking at 18 years.

Model 3: Adjusted for model 2+ birth weight z-score.

Model 4: Adjusted for model 3+ weight gain z-score birth to 20 months.

The calculation of the metabolic risk z-score is described in the methods section.

## Discussion

The study shows that birth-order is associated with increased body mass, adiposity and metabolic risk, according to conventional physiological and biochemical markers and after adjustment for multiple confounding variables associated with maternal and offspring socio-demographic status and health. The first-born effect was however tested in a cohort of young men and therefore further studies are required to establish with greater confidence the magnitude of the effect in other populations, and the potential variability in populations living industrialised and non-industrialised settings which may be exposed to different dietary and lifestyle factors.

A birth order effect on adiposity was observed in another cohort of young men aged 20 years, where first-borns had a 4-fold increase in the risk of excess adiposity compared to later-borns[17]. Other studies have also associated first-born status with growth differences in early life [10]. However, the metabolic implications of such greater adiposity have not previously been addressed and a formal comparison with our data cannot be attempted.

This is therefore the first study to investigate the long-term consequences of birth order on metabolic risk. The key strength of the study is the use of several markers of disease risk, and the representativeness of the cohort study for a young adult male population. In addition, when the estimates were adjusted for maternal smoking and socioeconomic status in childhood, a significantly reduced birth weight and greater infant weight gain in the first-borns was observed. However, a life-course epidemiological approach should be applied for the interpretation of the effects of birth order on metabolic risk, to account for other factors that might confound or explain some of the results (for example, family size, puberty, maternal and individual psychosocial stress).

The relationship between first-born status and metabolic risk found in this study is likely to be mediated by early growth patterns. Ong et al.[10] found in the ALSPAC cohort that first-borns had lower mean birth weight than later-borns (∼200 g) when controlling for smoking, gestational age and nutrition. The same analysis investigated growth patterns from birth to 5 years and found that first-born children became significantly heavier and taller children compared to later-borns[10]. The current evidence suggests that these two phenotypic growth patterns increase the risk of excess adiposity in children and adults as well as the risk of developing cardiovascular and metabolic disorders later in life (thrifty phenotype hypothesis) [27], [28]. This hypothesis is supported in our analysis, as associations between birth order and metabolic risk in the Brazilian cohort lost significance when early growth patterns were taken into account. Our analysis suggests that low birth weight does not itself explain the increased metabolic risk associated with birth order. Rather, rapid post-natal weight gain appears most important, although such rapid growth is itself a response to low birth weight. Broadly similar growth patterns have been linked to the occurrence of type 2 diabetes[29] and coronary events in adults[30].

The lower birth weight of first-borns can be attributed to materno-fetal physiological interactions. Following implantation, cells from the outer layer of the blastocyst, known as trophoblast, invade the maternal endometrium and alter the structure of the arteries that transfer blood to the placenta[31]. Such modification decreases maternal resistance and increases placental blood flow. These changes then impact on the placental dynamics of subsequent pregnancies[32], such that second-born neonates are well known to have higher average birth weight than first-borns. Dunger et al.[13] suggested that first-born children have higher glucose levels compared to later-borns, an effect most likely due to the combined effect of insulin resistance due to the increased adiposity and to the possible in utero programming of the insulin glucose axis[33]. Thus, the increased adult body weight and adiposity of first-borns is likely to be induced at least in part by the maternal constraint of intra-uterine growth. However, other mechanisms may also be important. There is preliminary evidence in animals [34] and in humans [35], [36] that the novel experience of the first pregnancy could raise the level of apprehension in primigravid women, thereby potentially affecting the growth of the foetus via modulation of the vascular and endocrine functions of the feto-placental unit [37], [38]. Maternal emotional stress is an established risk factor for low birth weight, intrauterine growth retardation, preterm delivery and still-birth[39], [40], [41]. Specifically, circadian cortisol secretion pattern appears to be distinctive in primiparous women and an alteration of the hypothalamus-pituitary axis (HPA) function could modify maternal glucocorticoids levels and affect foetal development[42], [43], [44]. Possible mechanisms for birth-order effects on foetal growth merit further research.

Our findings contribute to understanding of the early origins of adult disease. Our data show that a demographic factor relevant to all human populations can generate variability in both early growth and later metabolic risk. These findings also have important implications for understanding the increasing prevalence of the metabolic syndrome worldwide, where many populations are undergoing demographic change in response to economic development. Globally, there is a trend towards lower fertility rate, such that increasing proportion of individuals will be first-borns. In Brazil, for example, the average number of children per women (total fertility rate) dropped from 6.0 in 1960 to 1.8 currently.

Between- and within-country comparisons of trends in the prevalence of the metabolic syndrome may incorrectly attribute birth order effects to environmental factors. This has implications for monitoring the efficacy of public health campaigns aimed at reducing the prevalence of degenerative diseases, and also for the projection of future treatment costs. The public health implication of our findings is that the increased metabolic risk of first-borns is likely to derive from an interaction between their lower birth weight and conditions favouring rapid post-natal growth. Our findings therefore have implications for the optimal nutritional management of individual infants.

However, a number of questions still merit attention. For example, studies should describe in more detail the growth patterns that appear to lead to elevate metabolic risk, and identify the optimal time periods for intervention. Studies should also clarify the relative contribution of different possible underlying mechanisms (growth patterns, psychological factors) to the effects that we observed in these samples. Third, more research is required to establish the magnitude of the effect, whether it is similar in men and women, and whether it amplifies with age, as adverse metabolic profile consolidates. In these samples of young adults, the magnitude of the effect was relatively small, but degenerative diseases are expressed primarily from middle age and early-life effects tend to become more important through adulthood.
